# Methemoglobinemia in critically ill patients during extended hemodialysis and simultaneous disinfection of the hospital water supply

**DOI:** 10.1186/cc8128

**Published:** 2009-10-12

**Authors:** Martin Johannes Bek, Sven Laule, Christine Reichert-Jünger, Rainer Holtkamp, Michael Wiesner, Cornelius Keyl

**Affiliations:** 1 Dialysis Centre Bad Krozingen, Heart Centre Bad Krozingen, Suedring 15, 79189 Bad Krozingen, Germany; 2 Department of Anesthesiology, Heart Centre Bad Krozingen, Suedring 15, 79189 Bad Krozingen, Germany; 3 Department of Heart Surgery, Heart Centre Bad Krozingen, Suedring 15, 79189 Bad Krozingen, Germany

## Abstract

**Introduction:**

To evaluate the cause of methemoglobinemia in patients undergoing extended daily hemodialysis/hemodiafiltration we analyzed the relationship between methemoglobinemia and the water disinfection schedule of the hospital.

**Methods:**

We reviewed all arterial blood gas analyses, obtained over a one-year period, in patients undergoing extended hemodialysis/hemodiafiltration, and compared the methemoglobin concentrations obtained on the days when the water supply was disinfected, using a hydrogen peroxide/silver ion preparation, with data measured on disinfection-free days.

**Results:**

The evaluation of 706 measurements revealed a maximum methemoglobin fraction of 1.0 (0.8; 1.2) % (median and 25^th^; 75^th ^percentiles) during hemodialysis/hemodiafiltration on the disinfection-free days. The methemoglobin fraction increased to 5.9 (1.3; 8.4) % with a maximal value of 12.2% on the days of water disinfection (*P *< 0.001 compared to disinfection-free days). Spot checks on hydrogen peroxide concentrations in the water supply, the permeate, and the dialysate, using a semi-quantitative test, demonstrated levels between 10 and 25 mg/l during water disinfection.

**Conclusions:**

Our results demonstrate that even a regular hospital water disinfection technique can be associated with significant methemoglobinemia during extended hemodialysis. Clinicians should be aware of this potential hazard.

## Introduction

Extended daily hemodialysis/hemodiafiltration has been established as an effective renal replacement therapy in severely ill patients. Here we report cases of methemoglobinemia occurring intermittently during extended hemodialysis/hemodiafiltration in patients treated at a cardiac surgery intensive care unit (ICU). As common causes of methemoglobinemia could not be identified, we reviewed all arterial blood gas analyses obtained over a one-year period during extended hemodialysis/hemodiafiltration and analyzed the relation between methemoglobinemia and the water disinfection schedule of the hospital.

## Materials and methods

Data obtained in patients undergoing daily extended hemodialysis/hemodiafiltration between 1 February 2008 and 31 January 2009 were analyzed retrospectively. In view of the retrospective and descriptive character of the study, the local ethics committee waived the need for obtaining consent and the need for approval of the study.

Daily extended hemodialysis/hemodiafiltration (5 to 10 hours) was performed using AK200S dialysis machines and WRO300 H portable reverse osmosis systems (Gambro, Hechingen, Germany) connected to the hospital water supply. Substitution fluid was produced online, using a two-stage ultra-filtration system, and administered at a rate of 70 ml/min. Depending on the dialysis method, either FX60 or FX800 dialysers (Fresenius Medical Care, Bad Homburg, Germany) were used at blood flow rates of 200 ml/min. Dialysate flow rates were kept at 325 ml/min. The eliminated ultra-filtration volume ranged from 50 to 500 ml/h according to the clinical status of the patients. Standard anticoagulation was performed using unfractionated heparin or citrate.

After having identified the relation between the disinfection procedure and methemoglobinemia in February 2009, carbon filters (Wolftechnik Filtersysteme, Weil der Stadt, Germany) were interposed in the water supply of the portable reverse osmosis system.

Disinfection of the hospital water supply was performed using a hydrogen peroxide/silver ion preparation (Sanosil DGHM Universal-Desinfektion, Sanosil AG, Hombrechtikon, Switzerland). Water disinfection was performed at a stationary water-processing unit. The disinfectant was added proportionally to the main water flow using a digital dosage pump (Oxi-Des, Environ, Eschbach, Germany) over a time period of six to eight hours. The initial dosage was about 10 mg disinfectant per liter of water (mg/L), and was adjusted to maintain a concentration of 10 mg/L hydrogen peroxide during the disinfection period, measured at the peripheral water outlets (Peroxid-test, Merck, Darmstadt, Germany). Water disinfection was routinely performed bimonthly. Additionally, treatment was undertaken when construction work on the water supply had been performed. Due to frequent construction work, water disinfection was performed on four to six days per month during the analyzed time period.

Measurements of hydrogen peroxide concentrations in the permeate and dialysate were performed using semi-quantitative test strips with a sensitivity range from 0.5 to 25 mg/L (Quantofix Peroxid 25, Macherey-Nagel, Düren, Germany). The hemoglobin concentration and the fraction of methemoglobin were determined by CO-oximetry (ABL 825 flex, Radiometer Medical ApS, Brønshøj, Denmark). The data were obtained from the electronic patient database (Metavision, IMDsoft, Tel Aviv, Israel). The blood gas analysis with the highest methemoglobin fraction, obtained during each dialysis session, was taken for final analysis. The values of the hemoglobin concentration and methemoglobin fraction, obtained on the days when the water supply was disinfected, were compared with data measured on disinfection-free days. Statistical analysis was performed using commercially available software (SPSS for Windows 12.0.1., SPSS Inc., Chicago, IL, USA). Normal distribution of data was evaluated by visual assessment of the histograms and the probability plots (Q-Q plots). Data are reported in the case of normal distribution as mean ± standard deviation, otherwise as median with 25^th ^and 75^th ^percentiles. Differences between data, obtained on days when the water supply was disinfected and on disinfection-free days, were compared using non-parametric (Mann-Whitney U-test) or parametric tests (two-tailed Student's t test for unpaired data), as appropriate. *P *< 0.05 was regarded as statistically significant.

## Results

In 52 patients (27 male, 25 female, mean age 73 years, range 52 to 89 years, mean weight 77 ± 17 kg, mean height 167 ± 9 cm), 241 sessions of either extended hemodialysis or hemodiafiltration were performed between 1 February 2008 and 31 January 2009 with a mean duration of 466 ± 138 minutes. Of these dialysis sessions, 34 were performed on days when the hospital water supply was disinfected. During dialysis, 706 arterial blood gas analyses were carried out, 89 of them on the days of water disinfection.

The results of the methemoglobin measurements are presented in Table 1. The evaluation of the 241 measurements, taken for final analysis, revealed a significantly increased methemoglobin fraction during hemodialysis/hemodiafiltration on the days of water disinfection, compared with disinfection-free days, with a maximal value of 12.2%. In 21 dialysis sessions, performed on days with water disinfection, the methemoglobin fraction increased above 2%. Also, all methemoglobin values greater than 2.0% (n = 22) were measured on days of water disinfection, except for one (methemoglobin fraction 2.2%). The mean hemoglobin concentration of patients during extended hemodialysis/hemodiafiltration was slightly, but statistically significantly, lower on days with water disinfection (Table 1). Spot checks on the hydrogen peroxide concentrations in the water supply, the permeate and dialysate, using a semi-quantitative test, demonstrated levels between 10 and 25 mg/l during water disinfection. Control measurements before and after extended hemodialysis/hemodiafiltration did not indicate hydrogen peroxide, thus excluding the formation of hydrogen peroxide in the circuit of the dialysis machine independently of the water disinfection procedure.

**Table 1:** Maximum methemoglobin fraction and hemoglobin concentration during each session of extended hemodialysis/hemodiafiltration between 1 February 2008 and 31 January 2009

	Extended hemodialysis/hemodiafiltration on days with water disinfection	Extended hemodialysis/hemodiafiltration on disinfection-free days	P
Number of sessions	34	207	

Methemoglobin fraction (%)	5.9 (1.3; 8.4)	1.0 (0.8; 1.2)	< 0.001

Hemoglobin concentration (g/dl)	9.8 ± 0.9	10.3 ± 1.1	0.01

Data are reported as median (25^th^; 75^th ^percentiles) or as mean ± standard deviation.

The review of the administration of drugs with oxidizing properties (local anesthetics, nitroglycerine, sulfamethoxazole) revealed that one patient, who received all hemodialysis/hemodiafiltration sessions on disinfection-free days, had been treated with sulfamethoxazole. A nitroglycerine infusion had been administered in four patients during five sessions of hemodialysis/hemodiafiltration, all of which were on disinfection-free days. None of these treatments were associated with an increased methemoglobin fraction. Local anesthetics had not been administered to any of the patients.

All patients were clinically asymptomatic and showed no signs of suffering from hypoxia, such as newly occurring cardiac or cerebral symptoms, or hemolysis. Therefore, further laboratory tests, such as the determination of haptoglobin, were not carried out.

**Figure 1:**
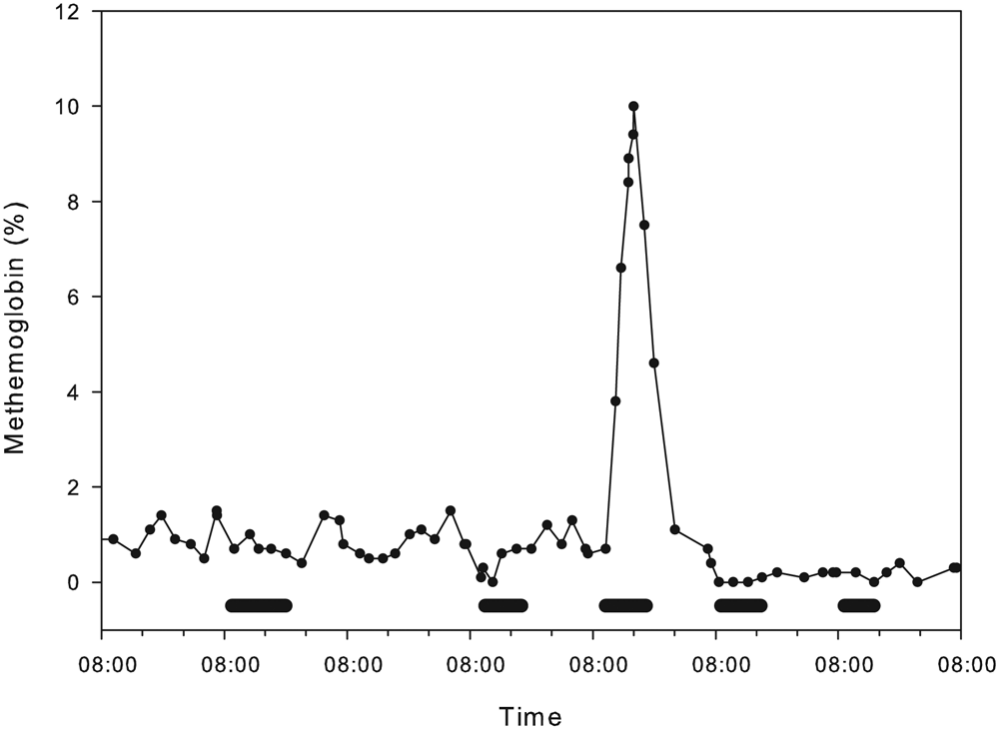
Methemoglobin fraction during a seven-day period in a patient undergoing five sessions of extended hemodiafiltration during that time (marked as horizontal bar).  On the fifth day, (i.e., during the third session of dialysis), disinfection of the hospital water supply was performed, accompanied by an increase in the methemoglobin fraction.

A sequence of all methemoglobin values obtained during a seven-day period in one patient is displayed in Figure 1. Disinfection of the water supply on the fifth day was associated with a marked increase in the methemoglobin concentration.

Starting in February 2009, a carbon filter was routinely interposed in the water supply of the portable reverse osmosis system. Since that time, we have not observed either hydrogen peroxide in the permeate and dialysate or increases in the methemoglobin fraction. The maximum methemoglobin fraction was 1.0%, 1.4%, and 1.2% in three patients undergoing extended hemodialysis/hemodiafiltration on days with water disinfection, and the hemoglobin concentration was 11.0 g/dl, 9.3 g/dl, and 9.9 g/dl, respectively. Hydrogen peroxide was not detectable in the permeate and dialysate, whereas its concentration was 10 mg/l at the peripheral water outlet.

## Discussion

The retrospective evaluation of arterial blood gas analyses demonstrated a constant association between hospital water disinfection, using a hydrogen peroxide/silver ion preparation, and the occurrence of methemoglobinemia during extended hemodialysis/hemodiafiltration in critically ill patients. Adverse effects of water disinfection with hydrogen peroxide have only been described in a few case reports until now. Davidovits and colleagues observed an association between methemoglobinemia up to 11% and hemolysis in children undergoing dialysis after the water storage tank had been disinfected with hydrogen peroxide and not sufficiently washed out [1]. Gordon and colleagues reported on children who developed hemolysis due to contamination of the dialysis fluid with hydrogen peroxide [2].

A systematic association between methemoglobinemia during dialysis and the water disinfection protocol of a hospital has not been described before. We observed methemoglobinemia in the context of a regularly performed water disinfection technique using a hydrogen peroxide/silver ion preparation. We could not find evidence for secondary methemoglobinemia due to other causes. Only a few patients were treated with drugs known to have oxidizing properties [3,4], and most of those treatments were performed on disinfection-free days. On these days, all patients, with the exception of one, had a methemoglobin fraction of 2% or lower. Apart from that single patient, all values above 2% occurred on days when the water supply was disinfected. Even if our patients were not clinically symptomatic according to the retrospective evaluation, impairment in the oxygen transport capacity should be strictly avoided in potentially cardiac-compromised patients, especially as hemoglobin concentrations are reduced in the post-operative setting. Furthermore, oxidative stress, as indicated by methemoglobinemia in our patients, is discussed as an aggravating factor for endothelial dysfunction and cell damage [5,6].

Methemoglobinemia has been reported to be associated with acute hemolytic anemia in previous case reports [1,2]. Due to the lack of clinical indication, specific laboratory tests for the diagnosis of hemolysis were not carried out in our patients. Therefore, it is not clear whether the slightly lower hemoglobin concentration during dialysis, on days when water disinfection had been performed, was caused by hemolysis.

The consequence of our findings was the interposition of carbon filters in the water supply of the portable reverse osmosis system. As a result, hydrogen peroxide has no longer been detected in the permeate and dialysate during water disinfection, and increases in the methemoglobin fraction have not been observed since.

### Limitations

As blood gas analyses had been originally undertaken for other purposes than the assessment of dyshemoglobinemia, it is not possible to evaluate the time course of methemoglobinemia during hemodialysis/hemodiafiltration systematically in our patients. Likewise, it is not possible to synchronize the exact time courses of hydrogen peroxide concentration in the hospital water supply and methemoglobinemia on the days of disinfection retrospectively. Due to the retrospective nature of our analysis and the absence of clinical signs, we did not perform analyses for hemolysis in our patients, as we would have done in a prospective study. Therefore, we cannot exclude the occurrence of a mild hemolysis due to hydrogen peroxide. Obviously, a prospective evaluation of this potentially harmful relations is contraindicated in the clinical setting.

## Conclusions

In conclusion, our results demonstrate that even a regular hospital water disinfection technique can be associated with significant methemoglobinemia during extended hemodialysis. Clinicians should be aware of this potential hazard.

## Key messages

• Disinfection of the hospital water supply, using a hydrogen peroxide/silver ion preparation, can be associated with a significant increase in the methemoglobin fraction in patients undergoing extended hemodialysis/hemodiafiltration.

• The use of reverse osmosis alone is not sufficient to provide adequate water contaminant removal, especially if the molecular weight is small. It should include the use of granulated carbon beds.

• Work on the hospital water supply, such as the addition of disinfectants, should not be undertaken without consultation of the medical staff in the renal and intensive care units.

## Abbreviations

ICU: intensive care unit.

## Competing interests

The authors declare that they have no competing interests.

## Authors' contributions

MJB contributed to the study design and wrote the manuscript. SL and RH revised the data and the manuscript critically. C R-J and MW collected the clinical data and drafted the manuscript. CK analyzed the data and wrote the manuscript.
